# Identifying the Local Influencing Factors of Arsenic Concentration in Suburban Soil: A Multiscale Geographically Weighted Regression Approach

**DOI:** 10.3390/toxics12030229

**Published:** 2024-03-21

**Authors:** Yuanli Zhu, Bo Liu, Gui Jin, Zihao Wu, Dongyan Wang

**Affiliations:** 1 School of Public Policy & Management, China University of Mining and Technology, Xuzhou 221116, China; 6268@cumt.edu.cn (Y.Z.); 11213957@cumt.edu.cn (B.L.); jingui@igsnrr.ac.cn (G.J.); 2 Research Center for Land Use & Ecological Security Governance in Mining Area, China University of Mining and Technology, Xuzhou 221116, China; 3 Research Center for Transformation Development and Rural Revitalization of Resource-Based Cities in China, China University of Mining and Technology, Xuzhou 221116, China; 4 College of Earth Sciences, Jilin University, Changchun 130061, China; wang_dy@jlu.edu.cn

**Keywords:** arsenic, multiscale geographically weighted regression, adaptative bandwidth, local influencing factor, spatial heterogeneity

## Abstract

Exploring the local influencing factors and sources of soil arsenic (As) is crucial for reducing As pollution, protecting soil ecology, and ensuring human health. Based on geographically weighted regression (GWR), multiscale GWR (MGWR) considers the different influence ranges of explanatory variables and thus adopts an adaptative bandwidth. It is an effective model in many fields but has not been used in exploring local influencing factors and sources of As. Therefore, using 200 samples collected from the northeastern black soil zone of China, this study examined the effectiveness of MGWR, revealed the spatial non-stationary relationship between As and environmental variables, and determined the local impact factors and pollution sources of As. The results showed that 49% of the samples had arsenic content exceeding the background value, and these samples were mainly distributed in the central and southern parts of the region. MGWR outperformed GWR with the adaptative bandwidth, with a lower Moran’s I of residuals and a higher R^2^ (0.559). The MGWR model revealed the spatially heterogeneous relationship between As and explanatory variables. Specifically, the road density and total nitrogen, clay, and silt contents were the primary or secondary influencing factors at most points. The distance from an industrial enterprise was the secondary influencing factor at only a few points. The main pollution sources of As were thus inferred as traffic and fertilizer, and industrial emissions were also included in the southern region. These findings highlight the importance of considering adaptative bandwidths for independent variables and demonstrate the effectiveness of MGWR in exploring local sources of soil pollutants.

## 1. Introduction

In recent years, soil arsenic (As) pollution caused by industrialization, agricultural production, and urbanization has become an important threat to soil ecology, food security, and human health [1,2,3]. The 2014 Chinese Soil Pollution Survey Bulletin reported that As is one of the main pollutants in the soil of arable land, mining areas, industrial parks, and both sides of trunk roads. High concentrations of As will disrupt soil microbial metabolism and plant growth, leading to the soil’s ecological deterioration and reduced food production [4,5]. More importantly, crops can absorb and enrich soil arsenic, posing a threat to human health [6,7]. Therefore, exploring the main sources and influencing factors of soil As is crucial for reducing soil arsenic pollution, protecting soil ecology, and ensuring human health.

The Kriging model [8], principal component analysis/cluster analysis [9], multiple linear regression (MLR) [10], geographical detectors [11,12], spatial lag models [13], and machine learning methods [14,15,16] have been used to explore the main influencing factors of As. These studies revealed that the main sources of As include mineral mining, industrial smelting, agricultural production, fossil energy combustion, traffic emissions, and some As-rich minerals (e.g., hutchinsonite and arsenopyrite). However, these global models assume that the relationship between the dependent and independent variables is the same in different subregions. This assumption may lead to the poor fitting performance of these models in areas with strong landscape heterogeneity [10,17]. Moreover, the global regression model can only reveal the average relationship between As and covariates throughout the entire study area and thus cannot provide data support for local As pollution treatment.

Geographically weighted regression (GWR) is a local spatial regression model that can determine the local impact factors of soil pollution at different locations [18]. It has been widely used to reveal the spatially heterogeneous relationships between soil pollutants and environmental variables [19,20,21]. The bandwidth is the core parameter of the GWR model, which reflects the influence range and scale effect of the independent variable on the dependent variable. However, GWR applies a fixed bandwidth to all covariates [22], which ignores that the impact scales of different covariates often vary in space. For example, climate factors and parent material typically control the spatial distribution of soil properties on a large scale, whereas human activities such as agricultural production and industrial emissions mainly affect the local soil [23,24]. To address this deficiency, Fotheringham et al. (2017) (Fotheringham, et al. [25]) proposed the multiscale GWR (MGWR) model, which fits an appropriate bandwidth for each explanatory variable. MGWR has been successfully used in the analysis of local impact factors on housing prices [26], the urban built environment [27], COVID-19 incidence rates [28], landslide susceptibility [29], ecological environment quality [30], and soil carbon storage [31]. Therefore, MGWR has great potential in determining the local pollution sources of As and proposing targeted pollution control measures.

Kuancheng District is located in the northeastern black soil zone of China and has a large area of fertile farmland. However, as a regional transportation hub, it undergoes rapid urbanization and industrialization, and its soil is at risk of arsenic pollution [32]. Therefore, this study aimed to evaluate the effectiveness of MGWR by comparing it with MLR and GWR, investigate the spatial non-stationary relationship between As and environmental variables, and explore the local impact factors and pollution sources of As.

## 2. Materials and Methods

### 2.1. Study Area

**Figure 1:**
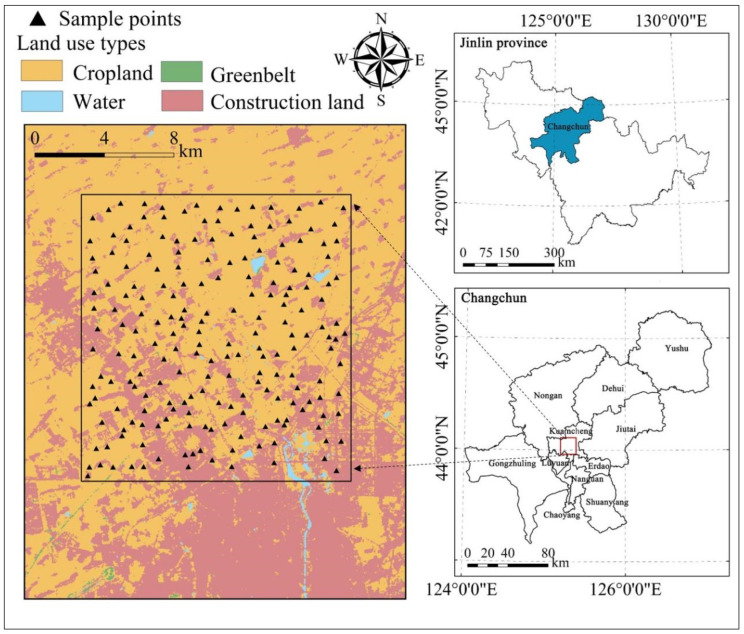
Location of study area and spatial distribution of sampling points.

The study area (43°57′~44°7′ N, 125°11′~125°24′ E) is located in the central southern part of Kuancheng District, Changchun City (Figure 1), and is typical suburban cultivated land affected by urbanization in the main grain-producing area of Northeast China. The research area is approximately 240 km^2^, with flat terrain and elevations ranging from 187 m to 246 m. Its climate is a typical continental monsoon climate, with an average annual precipitation of 550 mm and an average annual temperature of 4 °C. The research area mainly comprises cropland and construction land, with a small amount of forest, grassland, and water areas. The main lithology is a Holocene system, which contains sandstone and pebble sand wedge. The main soil types are meadow soil, phaeozem, and chernozeme, which are highly fertile.

### 2.2. Soil Sampling and Measurement

The urban–rural transition zone selected for the study includes different cultivated land utilization environments from urban to rural. Based on the land use map, the sampling locations were pre-determined to keep the sample density at 1 per km^2^, considering the land use type and the topographic conditions to ensure a uniform distribution of the sampling sites. Finally, 200 topsoil samples (0–20 cm) were taken in September 2017 (Figure 1). 

Each sample was composited by mixing three to five subsamples within a 1 m^2^ area. A stainless-steel spade was used for soil sampling and was washed with deionized water and wiped dry with paper towels between each use to avoid cross-contamination. The collected soil samples were air-dried to a constant weight, passed through a 2 mm sieve, and ground before being stored in the laboratory until soil As content analysis. 

For the analysis of total As, 0.25 g aliquots of the dried soil were digested in aqua regia (65% HNO_3_ to 37% HCl as 1:3) and analyzed using atomic fluorescence spectrometry (AFS200T, Skyray Instrument, Suzhou City, Jiangsu Province, China). Soil organic carbon (SOC) content was analyzed for all the samples following the Walkley–Black method [33], and soil organic matter (SOM) was determined by the van Bemmelen conversion factor along with the SOC concentration. Total nitrogen (TN) was determined using the Kjeldahl method. Soil pH was measured for a 1:2.5 soil/water ratio using a pH meter (PHS-3E, Leici, Shanghai, China). All sample analyses included batch replicates, reagent blanks, and standard reference materials from the National Research Center for Certified Reference Materials of China (GBW07424) to ensure analytical accuracy [34,35].

### 2.3. Sources and Pre-Processing of Environmental Variables

A total of 14 possible explanatory variables were selected from two aspects (Table 1) [36,37]: (i) possible sources of As, including distance from an industrial enterprise (Dis_IE), road density (RD), population density (PD), land use types (LU), total nitrogen (TN), total phosphorus (TP), and soil types (ST); (ii) migration-related factors of As, including clay content, silt content, pH, soil organic matter (SOM), elevation, and topographic wetness index (TWI).

**Figure 2:**
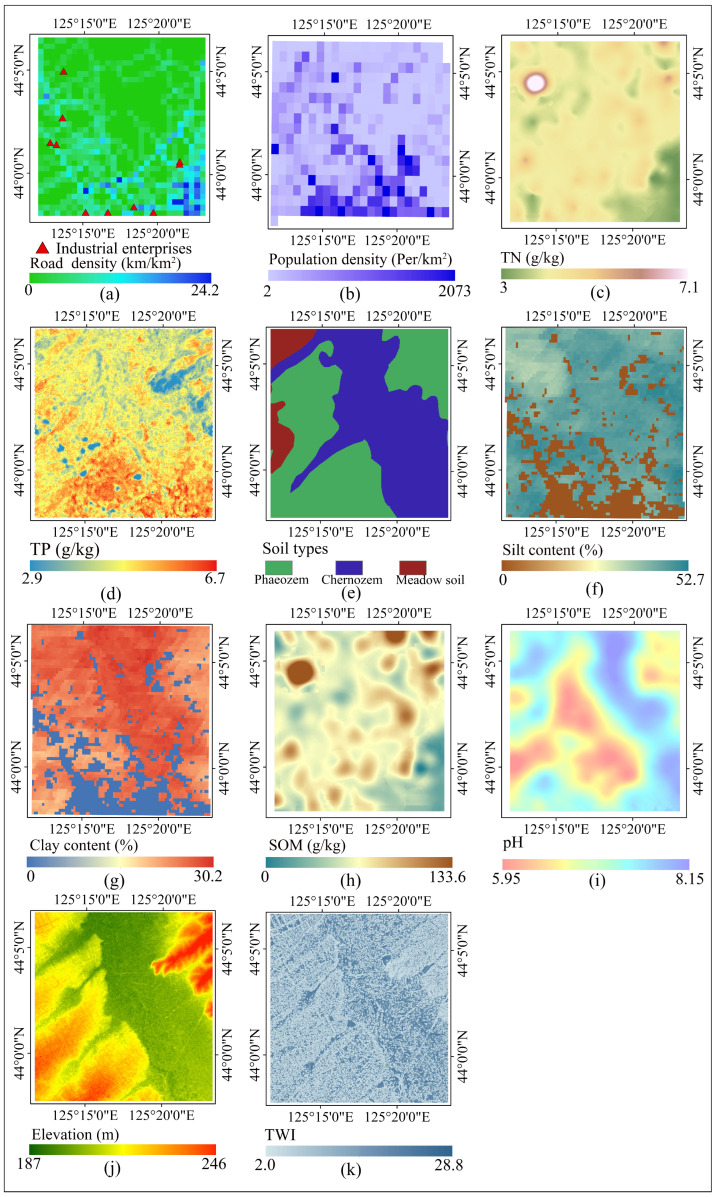
Spatial distribution maps of possible explanatory variables. (**a**) Spatial distribution map of industrial enterprises and road density. (**b**) Spatial distribution map of population density. (**c**) Spatial distribution map of total nitrogen. (**d**) Spatial distribution map of total phosphorus. (**e**) Spatial distribution map of soil types. (**f**) Spatial distribution map of silt content. (**g**) Spatial distribution map of clay content. (**h**) Spatial distribution map of soil organic matter content. (**i**) Spatial distribution map of pH. (**j**) Spatial distribution map of elevation. (**k**) Spatial distribution map of topographic wetness index. TN: total nitrogen; TP: total phosphorus; SOM: soil organic matter content; TWI: topographic wetness index.

We crawled the locations of industrial enterprises in the study area from Amap, as shown in Figure 2a. We calculated the distance from each sample point to the nearest industrial enterprise (Dis_IE). The land use type was derived from the China Land Cover Dataset (https://zenodo.org/, accessed on 1 September 2023), with a resolution of 30 m. The main land use types in the research area are cropland and construction land, as well as a small number of water bodies and a greenbelt (Figure 1). Road data were obtained from OpenStreetMap (https://www.openstreetmap.org/, accessed on 1 September 2023), including highways, city roads, suburban rural roads, and road density, which were determined using a 1 km raster, as shown in Figure 2a. The population density data were derived from LandScan Global Population Data (https://landscan.ornl.gov/, accessed on 1 September 2023), with a resolution of 1 km, as shown in Figure 2b. Total nitrogen and phosphorus and soil type are agricultural As sources. Total nitrogen was measured in the laboratory, and Figure 2c was obtained by ordinary Kriging interpolation with a resolution of 30 m. The total phosphorus from the National Earth System Science Data Center of China (http://soil.geodata.cn/ztsj.html, accessed on 1 September 2023), with a resolution of 90 m, is shown in Figure 2d. The soil type represents the natural source of As. The soil type data were obtained from the Chinese Resource and Environment Science and Data Center (https://www.resdc.cn/, accessed on 1 September 2023) with a resolution of 1 km. Soil types in the study area include phaeozem, chernozem, and meadow soil, as shown in Figure 2e.

The clay and silt contents were collected from SoilGrids (https://soilgrids.org/, accessed on 1 September 2023) with a resolution of 250 m. The silt content ranges from 0 to 52.7%, and the clay content ranges from 0 to 30.2%, as shown in Figure 2f,g, respectively. The data sources of SOM and pH are the same as that of total nitrogen, which was measured in the laboratory. The raster maps were obtained by Kriging interpolation with a resolution of 30 m, as shown in Figure 2h,i. The elevation data were obtained from NASA’s Earthdata website (https://nasadaacs.eos.nasa.gov/, accessed on 1 September 2023) with a resolution of 12.5 m, as shown in Figure 2j. The TWI was calculated based on the elevation data with a resolution of 30 m (Figure 2k). The ArcGIS software platform was used to complete all the environmental variable grid statistics, distance calculation, and spatial interpolation pre-processing work (version 10.7).

### 2.4. Spatial Local Regression Model

#### 2.4.1. GWR

GWR is an effective local spatial regression model [18,38]. It assumes that the relationship between dependent variables and covariates has spatial heterogeneity and thus establishes local linear regression equations at each sampling point. The formula of the GWR of the *i*th point with the coordinates $\left(u_{i},v_{i}\right)$ is as follows:

(1) $$y_{i}=\beta_{0}\left(u_{i},v_{i}\right)+\sum_{j=1}^{m}\beta_{j}\left(u_{i},v_{i}\right)x_{i,j}+\varepsilon_{i},$$

where $y_{i}$ is the dependent variable, $x_{i,j}$ is the *j*th explanatory variable, $\beta_{j}\left(u_{i},v_{i}\right)$ is the corresponding coefficient of $x_{i,j}$, $\beta_{0}\left(u_{i},v_{i}\right)$ is a constant term, and $\varepsilon_{i}$ is the stochastic error term. 

The bandwidth is the kernel function’s coverage range and is the GWR model’s core parameter. It determines the parameter estimation of the local regression equation using samples within a certain spatial range based on the scale effect of the independent variable’s influence on the dependent variable. An excessive bandwidth can lead to excessive bias in regression parameter estimation, whereas too small a bandwidth can lead to excessive variance in regression parameter estimation. Therefore, determining the optimal bandwidth size is crucial. However, GWR applies a fixed bandwidth to all covariates, which ignores that the impact scales of different covariates often vary in space. This assumption results in a biased estimation of regression parameters. In addition, GWR cannot robustly deal with parameter instability caused by outliers, multicollinearity, and spatial autocorrelation [39].

#### 2.4.2. MGWR

To address this deficiency, Fotheringham et al. (2017) (Fotheringham, Yang and Kang [25]) proposed the MGWR model. MGWR relaxes the assumption that all modeling processes are conducted on the same spatial scale. It adopts adaptative bandwidths for all explanatory variables to describe the scale effects of different covariates on the dependent variable. Thus, this model can reduce over-fitting with adaptative bandwidths and mitigate the concurrency of GWR [40]. The formula of MGWR is as follows:

(2) $$y_{i}=\beta_{0}\left(u_{i},v_{i}\right)+\sum_{j=1}^{m}\beta_{bw,j}\left(u_{i},v_{i}\right)x_{i,j}+\varepsilon_{i},$$

where bw in $\beta_{bw,j}$ is the adaptative bandwidth of $x_{i,j}$, and the meanings of other parameters are the same as those in Formula (1). When estimating local parameters, MGWR first sets the parameters of the GWR model as the initial parameters using the weighted least-squares method. Then, it uses the back-fitting algorithm to optimize the model parameters. Given the uneven distribution of sampling points, an adaptive bi-square kernel function and the Akaike information criterion (AIC) were used to obtain the optimal bandwidth of each explanatory variable.

This study used MLR, GWR, and MGWR models to explore the relationships between As and the explanatory variables. Their modeling and parameter estimation were conducted using SPSS (version 24.0) and MGWR software (version 2.2). Before modeling, the Moran’s I value of the As dataset was calculated using Geoda software (version 1.16) [41]. The use of GWR and MGWR spatial models is only meaningful if Moran’s I is significant. The log-likelihood, AIC, determination coefficient (R^2^), and residual Moran’s I were used to evaluate the performance of the three models. A well-performing model has a low AIC and residual Moran’s I and high log-likelihood and R^2^ values [42].

## 3. Results

### 3.1. Descriptive Statistics of As 

Table 2 exhibits the descriptive statistics results of As, TN, SOM, and pH. The As content ranged from 6.239 mg/kg to 15.966 mg/kg. Its mean and median values were 10.241 mg/kg and 10.088 mg/kg, respectively, close to the local background value of As (i.e., 10.25 mg/kg) [34]. The mean values of TN, SOM, and pH were 1.495 g/kg, 26.45 g/kg, and 7.085, respectively. The coefficients of variation of the four soil properties were 18.3%, 39.8%, 45.5%, and 13.2%.

Table 3 shows the one-way ANOVA results of soil and land use types. The mean As content among different soil and land use types was different, but they did not pass the F test (*p* > 0.05). The least significant difference test results showed that the mean As content of meadow soil was higher than that of black soil and significantly higher than that of chernozem. The mean As content of construction land was higher than that of cropland. These results indicated that soil and land use types affect the As content.

### 3.2. Spatial Distribution and Pollution Levels of As

**Figure 3:**
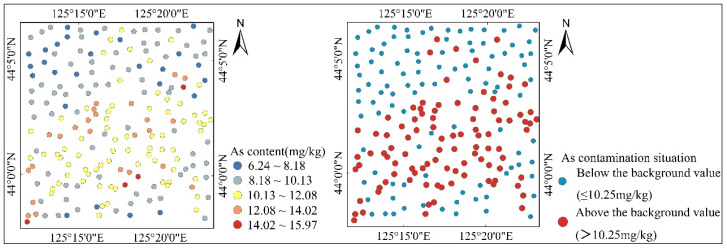
Spatial distribution maps of As content and contamination situation.

Figure 3 shows the spatial distribution of As content. The As content was higher in the central southern part of the study area, which is close to residential and industrial areas. It is lower in the northern part of the study area, which is close to agricultural areas. The As content in 49% of the samples exceeded the background value (10.25 mg/kg) [34]. However, none reached the risk screening value recorded in “Soil environmental quality—Risk control standard for soil contamination of agricultural land and development land” (Chinese GB36600-2018 [43] and GB15618-2018 [44]), indicating that agricultural production is still within a safe range. These samples are mainly distributed in the central and southern parts of the study area.

### 3.3. Model Comparison

**Figure 4:**
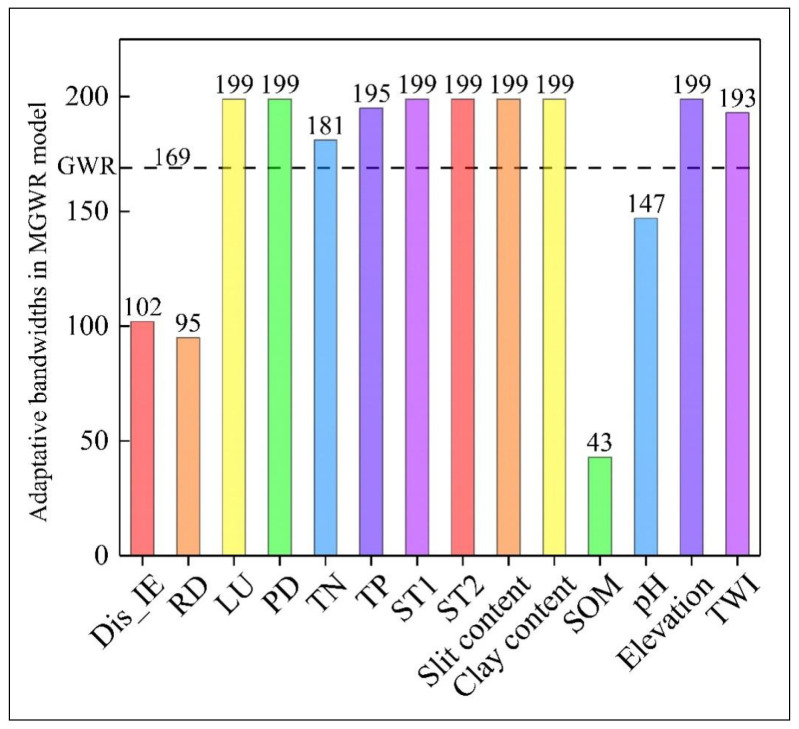
Optimal bandwidths of explanatory variables for GWR and MGWR models. Dis_IE: distance from an industrial enterprise; RD: road density; LU: land use type; PD: population density; TN: total nitrogen; TP: total phosphorus; ST1, 2: dummy variable for soil type; SOM: soil organic matter content; TWI: topographic wetness index.

The Moran’s I value of As content was 0.477 (*p* < 0.01), which confirms that As content has spatial autocorrelation and should be fitted via the GWR and MGWR models. Figure 4 exhibits the optimal bandwidth of explanatory variables for GWR and MGWR models. The optimal bandwidth in GWR was 169. In MGWR, the optimal bandwidths of land use, TN, TP, soil type, clay and silt contents, elevation, and TWI ranged from 181 to 199, which were close to the whole study area. SOM had the smallest optimal bandwidth of 43, followed by road density, Dis_IE, and pH. These results demonstrate the importance of considering an adaptative bandwidth in the MGWR model.

**Figure 5:**
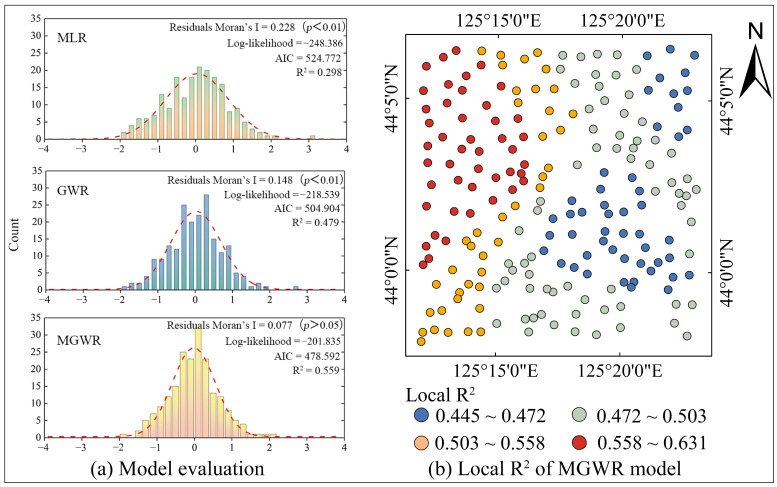
Results of model evaluation and spatial distribution map of local R^2.^ (**a**) Results of model evaluation. (**b**) Results of spatial distribution map of local R^2^.

Figure 5a displays the model evaluation results of MLR, GWR, and MGWR. MGWR outperformed GWR and MLR, with a higher R2 and log-likelihood and lower AIC and Moran’s I of residuals. In particular, the residuals’ Moran’s I of MLR and GWR was significant, whereas the residuals’ Moran’s I of MGWR was not significant. These results indicated that MGWR was the optimal model. Thus, we only show the regression results of MGWR in the following sections. 

The local R^2^ of the MGWR model ranged from 0.445 to 0.631. Its spatial distribution is shown in Figure 5b. The local R^2^ was highest in the western region and decreased from west to east.

### 3.4. Spatial Distribution of MGWR Regression Coefficients

Table 4 shows the descriptive statistics of the standardized regression coefficients of the explanatory variables for the MGWR model. The absolute value of the mean standardized coefficient is the highest for road density, which was 1.521, followed by silt content, clay content, TN, and TP, which were 0.383, 0.305, 0.286, and 0.219, respectively. The absolute values of the mean standardized coefficient of other factors were relatively small, all below 0.200. The significant number of explanatory variables was also counted. The silt and clay contents were substantial at all 200 locations. The RD, land use, TP, and pH were significant at 116 to 198 locations. Dis_IE, TN, and SOM were only significant at several locations, whereas other variables were not significant at any locations.

**Figure 6:**
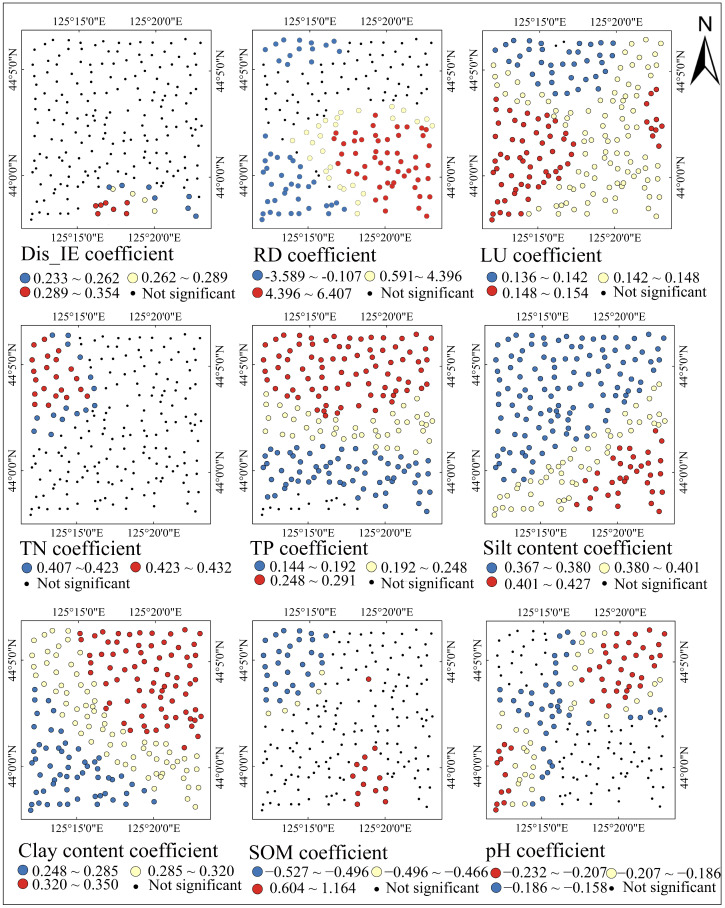
Spatial distribution maps of significant standardized coefficients for the MGWR model. Dis_IE: distance from the industrial enterprise; RD: road density; LU: land use type; TN: total nitrogen; TP: total phosphorus; SOM: soil organic matter content.

Figure 6 displays the spatial distribution of significant standardized coefficients. Variables that were not significant at any location are not shown. Dis_IE was only significant at some points in the southeastern part of the study area. The highest absolute values of RD, silt content, and SOM all occurred in the southeast of the region and decreased along the southeast–northwest direction. However, the RD and SOM were not significant in the central region. The coefficients of TN and TP showed a decreasing trend from north to south, but TN was only significant in the northwest of the region. The spatial distribution of the clay content coefficient was symmetrical with the spatial distribution of silt content. It decreased in the northeast–southwest direction. The coefficient values of pH and LU had relatively small spatial differences, with an overall trend of high values in the west and east.

To sum up, the coefficients of industrial and traffic factors were higher in the southeastern part of the study area, whereas the coefficients of agricultural factors were higher in the northern part of the region. The coefficients of soil environmental factors varied less spatially in the study area.

### 3.5. Local Influencing Factors and Sources of As

**Figure 7:**
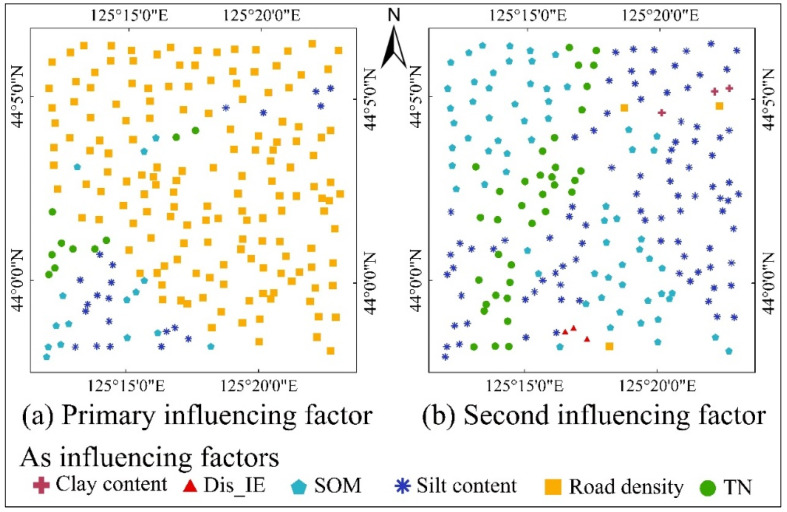
Spatial distribution maps of main influencing factors at each sampling point. (**a**) Spatial distribution map of the primary influencing factor. (**b**) Spatial distribution map of the second influencing factor. Dis_IE: distance from the industrial enterprise; SOM: soil organic matter content; TN: total nitrogen.

The absolute values of the coefficients of each variable were sorted from highest to lowest, and the primary and secondary influencing factors for As content at each sampling point were determined (Figure 7). The primary influencing factor of As content for most points is road density. The primary influencing factor of As content for some samples in the southwestern region was SOM, silt content, or TN. The second influencing factor of As content for most points was SOM, silt content, or TN, whereas the second influencing factor of As content for only a small number of points was clay content, Dis_IE, or RD.

**Figure 8:**
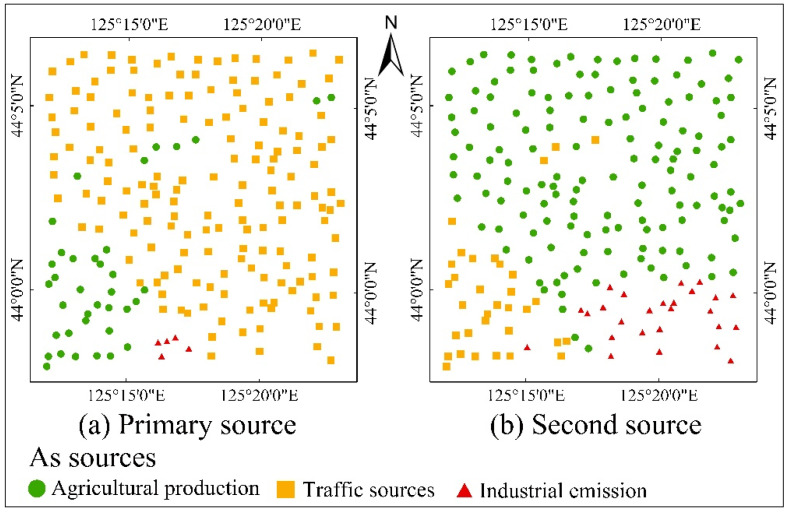
Spatial distribution maps of the main sources of As at each sampling point. (**a**) Spatial distribution map of the primary source. (**b**) Spatial distribution map of the second source.

Based on the relative importance of source-related variables, the main sources of As at each point were inferred and are exhibited in Figure 8. The traffic source characterized by the road density was the primary or secondary source of As for almost all locations. The agricultural source, represented by TN and TP, was the second source for most points and the primary source for a small number of points located in the southwest of the region. The primary or secondary source, with only a few points located in the southeast of the region, was industrial emissions, characterized by Dis_IE. 

To sum up, the main influencing factors and sources of As varied greatly at different locations. Traffic, fertilization, and industrial emissions were the primary sources of As in the study area, and soil environmental factors were crucial for soil As accumulation. 

## 4. Discussion

### 4.1. Impact of Environmental Variables on As Accumulation

Based on the mean absolute standardized coefficients, the road density, silt content, clay content, TN, and TP were the main influencing factors of As content, and traffic and agricultural sources were the main sources of As.

Kuancheng District is the transportation hub of Changchun City, with two railway stations, long-distance bus stations, and a high road density within the territory [32,34]. With rapid urbanization, the road density and residential car ownership in the study area are rapidly increasing. Given that road surface materials and vehicle components contain As, an increasing road density will lead to more As accumulation in soil [45]. Zechmeister et al. (2005) [46] found that friction between heavy-duty vehicle tires and the road is particularly conducive to the accumulation of pollutants such as As in roadside soil and moss. Many studies have observed arsenic pollution in the soil on both sides of the road [47,48,49,50]. In this study, we found that As was positively related to the road density, which is consistent with the research of Seker et al. (2022) (Seker, et al. [51]) and Qiao et al. (2022) (Qiao, et al. [52]).

Using arsenical animal feed and phosphorus fertilizers in agricultural production is another important source of soil arsenic [53,54,55,56]. For example, arsenic compounds such as roxarsone are commonly used as feed additives in livestock farming and enter the soil through livestock manure [57,58]. In addition, several studies found As in phosphate and compound fertilizers [59,60]. Using these feeds and fertilizers increases total nitrogen, total phosphorus, and arsenic contents in the soil. As a result, TN and TP showed a positive relation with As content.

The emissions from non-ferrous metal smelting and fossil fuel combustion are commonly considered the most important sources of arsenic pollution [61,62]. Arsenic diffuses outward with industrial waste gas and wastewater. As a result, As is often negatively correlated with the distance to a factory [63,64]. The present study also found such a relationship, but the mean absolute standardized coefficient of Dis_IE was relatively small. The reason may be that most enterprises in the research area are food processing, weaving, and clothing factories, while mining and metal smelting factories are fewer.

Soil properties, including pH, clay content, and silt content, affect As accumulation by affecting the adsorption/desorption of arsenic [65,66]. Specifically, the increase in pH causes the soil to carry more negative charges and repel arsenate, leading to the desorption of solid arsenic in the soil [67,68]. This increase will lead to an increase in available arsenic content and a decrease in soil arsenic content. As the soil particles become smaller, the specific surface area increases, and the charge density increases, which enhances the soil’s ability to adsorb arsenic [69,70]. As a result, pH was negatively related to As content, while clay and silt contents were positively related to As content. 

### 4.2. Scale Effect of Explanatory Variables

The results of MGWR show great differences in the optimal bandwidth for different explanatory variables, indicating that the adaptative bandwidth is the main reason that MGWR outperforms GWR. This result also reflects the significant differences in the range of influence of different explanatory variables on As, which may be related to the spatial heterogeneity and inherent properties of the explanatory variables.

Natural factors such as elevation, TWI, pH, clay content, and silt content have larger bandwidths, whereas road density and Dis_IE have smaller bandwidths. On the one hand, natural factors typically control the spatial distribution of soil attributes on a larger scale, whereas human activity factors typically affect soil spatial patterns on a smaller scale [23,24]. On the other hand, the number of local factories is small, and no heavily polluting industries are identified. Thus, the pollution impact range of industrial enterprises is relatively small.

The proper bandwidth of SOM was the smallest of all variables, only 43. The reason may be that farmers often adopt different cropping strategies. After long-term cultivation, the spatial dependence of SOM decreases, while spatial heterogeneity increases (Figure 2i) [71,72]. Therefore, the impact range of SOM on As is relatively small.

These results emphasize the importance of considering adaptative bandwidths for independent variables and confirm the effectiveness of MGWR.

### 4.3. Limitations

Although the 14 explanatory variables can explain 55.9% of As variation, some important factors were not included in the MGWR model due to data acquisition limitations. For example, iron minerals, aluminum oxides, and soil sulfur cycling all affect the adsorption/desorption of As [65,73,74]. In future research, combining parent materials, high-resolution lithology, and Fe, Al, and S contents may further enhance the R^2^ of the regression model. 

The MGWR model assumes that the relationship between As and a covariate was linear. However, several studies have used machine learning methods to reveal non-linear correlations between soil properties and covariates [14,15,16]. How to combine MGWR with machine learning to obtain an MGWR–machine learning model remains to be explored. 

## 5. Conclusions

This study explored the effectiveness of MGWR, revealed the spatial non-stationary relationship between As and environmental variables, and determined the local impact factors and pollution sources of As in the Kuangcheng District. The results showed that 49% of the samples had arsenic content exceeding the background value, and these samples were mainly distributed in the central and southern parts of the region. The optimal bandwidth of different variables varies greatly in the MGWR model. MGWR outperformed GWR and MLR, and its R^2^ reached 0.559. The MGWR model revealed spatially heterogeneous relationships between As and the explanatory variables. In particular, the road density, TN, clay, and silt content were primary or secondary influencing factors at most points, whereas Dis_IE and SOM were secondary influencing factors at only a few points. The main pollution sources of As were thus inferred as traffic and fertilizer, and industrial emission was also included in the southern region. These findings highlight the importance of considering adaptative bandwidths for independent variables and demonstrate the effectiveness of MGWR in exploring local sources of soil pollutants.

## Figures and Tables

**Table 1:** Possible influencing factors of As and their data sources.

Aspect	Possible Influencing Factor	Data Sources and Links
Possible sources of As	Distance from an industrial enterprise	Crawled data from Amap, POI data, https://map.amap.com, accessed on 1 September 2023
	Land use type	China Land Cover Dataset, Raster data (30 m), https://zenodo.org/, accessed on 1 September 2023
	Population density	LandScan Global Population Data, Raster data (1000 m), https://landscan.ornl.gov/, accessed on 1 September 2023
	Road density	Open Street Map, Vector data, https://www.openstreetmap.org/, accessed on 1 September 2023
	Total nitrogen	Measured in the laboratory
	Total phosphorus	National Earth System Science Data Center of China, Raster data (90 m), http://soil.geodata.cn/ztsj.html, accessed on 1 September 2023
	Soil type	Chinese Resource and Environment Science and Data Center, Raster data (1000 m), https://www.resdc.cn/, accessed on 1 September 2023
Migration-related factors of As	Clay and silt content	SoilGrids, Raster data (250 m), https://soilgrids.org/, accessed on 1 September 2023
	pH and SOM	Measured in the laboratory
	Elevation and topographic wetness index (TWI)	NASA’s Earth data website, Raster data (12.5 m), https://nasadaacs.eos.nasa.gov/, accessed on 1 September 2023

**Table 2:** Descriptive statistics of measured soil properties.

Soil Properties	Mean ± Std	Minimum	Median	Maximum	CV (%)
As (mg/kg)	10.241 ± 1.870	6.239	10.088	15.966	18.3
TN (g/kg)	1.495 ± 0.595	0.300	1.459	7.175	39.8
SOM (g/kg)	26.45 ± 12.03	3.76	25.35	138.0	45.5
pH	7.085 ± 0.937	4.81	7.31	9.05	13.2

CV: coefficient of variation.

**Table 3:** One-way ANOVA results of soil and land use types.

Soil Types	Mean As Content	Land Use Types	Mean As Content
Meadow soil	10.389 ± 1.593 ^a^	Construction land	10.593 ± 1.759 ^a^
Phaeozem	10.266 ± 2.106 ^ab^	Cropland	10.104 ± 1.890 ^a^
Chernozem	9.324 ± 1.920 ^b^		
F test: *p* > 0.05		F test: *p* > 0.05	

^a,b^ indicate significant differences via least significant difference test.

**Table 4:** Standardized regression coefficients of explanatory variables for the MGWR model.

Explanatory Variables	Mean	Std	Min	Median	Max	Number of Significant Results
Dis_IE	0.029	0.137	−0.171	0.011	0.354	18
RD	1.521	3.072	−3.589	0.547	6.407	126
LU	0.146	0.004	0.135	0.146	0.154	198
PD	−0.024	0.030	−0.073	−0.025	0.027	0
TN	0.286	0.113	0.105	0.299	0.432	36
TP	0.219	0.052	0.133	0.226	0.291	186
ST1	0.095	0.02	0.063	0.092	0.130	0
ST2	0.004	0.018	−0.027	0.002	0.035	0
Slit content	0.383	0.016	0.367	0.377	0.427	200
Clay content	0.305	0.030	0.248	0.308	0.350	200
SOM	0.031	0.402	−0.556	−0.023	1.164	47
pH	−0.164	0.046	−0.232	−0.171	−0.064	116
Elevation	0.045	0.037	−0.019	0.050	0.099	0
TWI	0.026	0.031	−0.031	0.026	0.075	0

Dis_IE: distance from an industrial enterprise; RD: road density; LU: land use type; PD: population density; TN: total nitrogen; TP: total phosphorus; ST1, ST2: dummy variable for soil type; SOM: soil organic matter content; TWI: topographic wetness index.

## Data Availability

Data are contained within the article.

## References

[1] Zhou C., Wang J., Wang Q., Leng Z., Geng Y., Sun S., Hou H. (2023). Simultaneous adsorption of Cd and As by a novel coal gasification slag based composite: Characterization and application in soil remediation. Sci. Total Environ..

[2] Siddiqui M.F., Khan Z.A., Jeon H., Park S. (2020). SPE based soil processing and aptasensor integrated detection system for rapid on site screening of arsenic contamination in soil. Ecotoxicol. Environ. Saf..

[3] Zecchin S., Wang J., Martin M., Romani M., Planer-Friedrich B., Cavalca L. (2023). Microbial communities in paddy soils: Differences in abundance and functionality between rhizosphere and pore water, influence of different soil organic carbon, sulfate fertilization, and cultivation time, and contribution to arsenic mobility and speciation. FEMS Microbiol. Ecol..

[4] Zhu T., Feng L., Cao C. (2023). Effects of arsenic on bioelectricity output and anode microbial community of soil microbial fuel cells in arsenic-petroleum hydrocarbon-contaminated soils. J. Chem. Technol. Biotechnol..

[5] Ivy N., Bhattacharya S., Dey S., Gupta K., Dey A., Sharma P. (2023). Effects of microplastics and arsenic on plants: Interactions, toxicity and environmental implications. Chemosphere.

[6] Golui D., Raza M.B., Roy A., Mandal J., Sahu A.K., Ray P., Datta S.P., Rahman M.M., Bezbaruah A. (2023). Arsenic in the Soil-Plant-Human Continuum in Regions of Asia: Exposure and Risk Assessment. Curr. Pollut. Rep..

[7] Rehman M.U., Khan R., Khan A., Qamar W., Arafah A., Ahmad A., Ahmad A., Akhter R., Rinklebe J., Ahmad P. (2021). Fate of arsenic in living systems: Implications for sustainable and safe food chains. J. Hazard. Mater..

[8] Muhammad A.M., Tang Z., Xiao T. (2022). Evaluation of the factors affecting arsenic distribution using geospatial analysis techniques in Dongting Plain, China. Front. Environ. Sci..

[9] Kun Z., Cai Y., Chen W., Peng P. (2023). Source identification and spatial distribution of heavy metals in soil of central urban area of Chongqing, China. Soil Sediment Contam..

[10] Zheng M., Luan H., Liu G., Sha J., Duan Z., Wang L. (2023). Ground-Based Hyperspectral Retrieval of Soil Arsenic Concentration in Pingtan Island, China. Remote Sens..

[11] Shi B., Cai K., Yan X., Liu Z., Zhang Q., Du J., Yang X., Luan W. (2023). Spatial Distribution and Migration Mechanisms of Toxic Elements in Farmland Soil at Nonferrous Metal Smelting Site. Water.

[12] Zeng J., Ke W., Deng M., Tan J., Li C., Cheng Y., Xue S. (2023). A practical method for identifying key factors in the distribution and formation of heavy metal pollution at a smelting site. J. Environ. Sci..

[13] Nigra A.E., Cazacu-De Luca A., Navas-Acien A. (2022). Socioeconomic vulnerability and public water arsenic concentrations across the US. Environ. Pollut..

[14] Jia X., Hou D. (2023). Mapping soil arsenic pollution at a brownfield site using satellite hyperspectral imagery and machine learning. Sci. Total Environ..

[15] Kumar S., Pati J. (2022). Assessment of groundwater arsenic contamination level in Jharkhand, India using machine learning. J. Comput. Sci..

[16] Kumar S., Pati J. (2023). Machine learning approach for assessment of arsenic levels using physicochemical properties of water, soil, elevation, and land cover. Environ. Monit. Assess..

[17] Yang L., Meng F., Ma C., Hou D. (2022). Elucidating the spatial determinants of heavy metals pollution in different agricultural soils using geographically weighted regression. Sci. Total Environ..

[18] Fotheringham A.S., Charlton M.E., Brunsdon C. (1998). Geographically weighted regression: A natural evolution of the expansion method for spatial data analysis. Environ. Plan. A.

[19] Li H., Fu P., Yang Y., Yang X., Gao H., Li K. (2021). Exploring spatial distributions of increments in soil heavy metals and their relationships with environmental factors using GWR. Stoch. Environ. Res. Risk Assess..

[20] Qu M., Liu H., Guang X., Chen J., Zhao Y., Huang B. (2022). Improving correction quality for in-situ portable X-ray fluorescence (PXRF) using robust geographically weighted regression with categorical land-use types at a regional scale. Geoderma.

[21] Ye M., Zhu L., Li X., Ke Y., Huang Y., Chen B., Yu H., Li H., Feng H. (2023). Estimation of the soil arsenic concentration using a geographically weighted XGBoost model based on hyperspectral data. Sci. Total Environ..

[22] Yu H., Fotheringham A.S., Li Z., Oshan T., Kang W., Wolf L.J. (2019). Inference in Multiscale Geographically Weighted Regression. Geogr. Anal..

[23] Lamichhane S., Kumar L., Wilson B. (2019). Digital soil mapping algorithms and covariates for soil organic carbon mapping and their implications: A review. Geoderma.

[24] Shary P.A. (2023). Environmental Variables in Predictive Soil Mapping: A Review. Eurasian Soil Sci..

[25] Fotheringham A.S., Yang W., Kang W. (2017). Multiscale Geographically Weighted Regression (MGWR). Ann. Am. Assoc. Geogr..

[26] Zhang Z., Li J., Fung T., Yu H., Mei C., Leung Y., Zhou Y. (2021). Multiscale geographically and temporally weighted regression with a unilateral temporal weighting scheme and its application in the analysis of spatiotemporal characteristics of house prices in Beijing. Int. J. Geogr. Inf. Sci..

[27] Xu J., Jing Y., Xu X., Zhang X., Liu Y., He H., Chen F., Liu Y. (2023). Spatial scale analysis for the relationships between the built environment and cardiovascular disease based on multi-source data. Health Place.

[28] Mansour S., Al Kindi A., Al-Said A., Al-Said A., Atkinson P. (2021). Sociodemographic determinants of COVID-19 incidence rates in Oman: Geospatial modelling using multiscale geographically weighted regression (MGWR). Sustain. Cities Soc..

[29] Li Y., Huang S., Li J., Huang J., Wang W. (2022). Spatial Non-Stationarity-Based Landslide Susceptibility Assessment Using PCAMGWR Model. Water.

[30] Wang T., Zhao M., Gao Y., Yu Z., Zhao Z. (2023). Analyzing Spatial-Temporal Change of Vegetation Ecological Quality and Its Influencing Factors in Anhui Province, Eastern China Using Multiscale Geographically Weighted Regression. Appl. Sci..

[31] Wen X., Zhang Z., Huang X. (2022). Heavy metals in karst tea garden soils under different ecological environments in southwestern China. Trop. Ecol..

[32] Yang Y., Wang D., Yan Z., Zhang S. (2021). Delineating Urban Functional Zones Using U-Net Deep Learning: Case Study of Kuancheng District, Changchun, China. Land.

[33] Nelson D.W. (1996). Total Carbon, Organic Carbon, and Organic Matter.

[34] Zhu Y., Wang D., Li W., Yang Y., Shi P. (2019). Spatial distribution of soil trace element concentrations along an urban-rural transition zone in the black soil region of northeastern China. J. Soils Sediments.

[35] Meng X. (1995). Study on Background Values of Soil Elements in Jilin Province.

[36] Abbas F., Hammad H.M., Ishaq W., Farooque A.A., Bakhat H.F., Zia Z., Fahad S., Farhad W., Cerda A. (2020). A review of soil carbon dynamics resulting from agricultural practices. J. Environ. Manag..

[37] Xu J., Xiao P. (2022). Influence factor analysis of soil heavy metal based on categorical regression. Int. J. Environ. Sci. Technol..

[38] Fotheringham A.S., Brunsdon C.F., Charlton M.E. (2002). Geographically Weighted Regression: The Analysis of Spatially Varying Relationships.

[39] Farber S., Páez A. (2007). A systematic investigation of cross-validation in GWR model estimation: Empirical analysis and Monte Carlo simulations. J. Geogr. Syst..

[40] Iyanda A.E., Osayomi T. (2020). Is there a relationship between economic indicators and road fatalities in Texas? A multiscale geographically weighted regression analysis. GeoJournal.

[41] Dray S., Legendre P., Peres-Neto P.R. (2006). Spatial modelling: A comprehensive framework for principal coordinate analysis of neighbour matrices (PCNM). Ecol. Model..

[42] Wu Z., Chen Y., Han Y., Ke T., Liu Y. (2020). Identifying the influencing factors controlling the spatial variation of heavy metals in suburban soil using spatial regression models. Sci. Total Environ..

[43] (2018). Soil Environmental Quality Risk Control Standard for Soil Contamination of Development Land.

[44] (2018). Soil Environmental Quality Risk Control Standard for Soil Contamination of Agricultural Land.

[45] Hiller E., Pilkova Z., Filova L., Jurkovic L., Mihaljevic M., Lacina P. (2021). Concentrations of selected trace elements in surface soils near crossroads in the city of Bratislava (the Slovak Republic). Environ. Sci. Pollut. Res..

[46] Zechmeister H.G., Hohenwallner D., Riss A., Hanus-Illar A. (2005). Estimation of element deposition derived from road traffic sources by using mosses. Environ. Pollut..

[47] Mama C.N., Igwe O., Ezugwu C.K., Ozioko O., Ugwuoke I.J. (2021). Statistical aproach to unravelling heavy metal contamination on sub-soils and roadside dust. Int. J. Environ. Anal. Chem..

[48] Mama C.N., Nnaji C.C., Igwe O., Ozioko O.H., Ezugwu C.K., Ugwuoke I.J. (2022). Assessment of heavy metal pollution in soils: A case study of Nsukka metropolis. Environ. Forensics.

[49] Davis H.T., Aelion C.M., Liu J., Burch J.B., Cai B., Lawson A.B., McDermott S. (2016). Potential sources and racial disparities in the residential distribution of soil arsenic and lead among pregnant women. Sci. Total Environ..

[50] Kondo M.C., Zuidema C., Moran H.A., Jovan S., Derrien M., Brinkley W., De Roos A.J., Tabb L.P. (2022). Spatial predictors of heavy metal concentrations in epiphytic moss samples in Seattle, WA. Sci. Total Environ..

[51] Seker M.E., Erdogan A., Korkmaz S.D., Kuplulu O. (2022). Bee pollens as biological indicators: An ecological assessment of pollution in Northern Turkey via ICP-MS and XPS analyses. Environ. Sci. Pollut. Res..

[52] Qiao Y., Wang X., Han Z., Tian M., Wang Q., Wu H., Liu F. (2022). Geodetector based identification of influencing factors on spatial distribution patterns of heavy metals in soil: A case in the upper reaches of the Yangtze River, China. Appl. Geochem..

[53] Hung C.-C., Lin H.-T., Chen C.-Y., Chen K.-Y., Lee T.-Y., Chiang C.-F. (2023). Estimating arsenic biotransfer factors from feed to chicken: A viable approach to animal feed risk assessment. Food Addit. Contam. Part A-Chem. Anal. Control Expo. Risk Assess..

[54] Fathi-Gerdelidani A., Towfighi H., Shahbazi K. (2022). Kinetic studies on arsenic release from geogenically enriched soils under oxidized and reduced conditions. J. Geochem. Explor..

[55] Zhang S., Li X., Chen K., Shi J., Wang Y., Luo P., Yang J., Wang Y., Han X. (2022). Long-term fertilization altered microbial community structure in an aeolian sandy soil in northeast China. Front. Microbiol..

[56] Zhao Z., Deng X., Zhang F., Li Z., Shi W., Sun Z., Zhang X. (2022). Scenario Analysis of Livestock Carrying Capacity Risk in Farmland from the Perspective of Planting and Breeding Balance in Northeast China. Land.

[57] Wang C., Ren G., Tan Q., Che G., Luo J., Li M., Zhou Q., Guo D.-Y., Pan Q. (2023). Detection of organic arsenic based on acid-base stable coordination polymer. Spectrochim. Acta Part A Mol. Biomol. Spectrosc..

[58] Wang X., Wu Q., Wang Z.-Z., Ma W.-J., Qiu J., Fan N.-S., Jin R.-C. (2023). Biotransformation-mediated detoxification of roxarsone in the anammox process: Gene regulation mechanism. Chem. Eng. J..

[59] Battaglia-Brunet F., Le Guedard M., Faure O., Charron M., Hube D., Devau N., Joulian C., Thouin H., Hellal J. (2021). Influence of agricultural amendments on arsenic biogeochemistry and phytotoxicity in a soil polluted by the destruction of arsenic-containing shells. J. Hazard. Mater..

[60] Islam M.S., Mostafa M.G. (2021). Influence of chemical fertilizers on arsenic mobilization in the alluvial Bengal delta plain: A critical review. AQUA-Water Infrastruct. Ecosyst. Soc..

[61] Mpewo M., Kizza-Nkambwe S., Kasima J.S. (2023). Heavy metal and metalloid concentrations in agricultural communities around steel and iron industries in Uganda: Implications for future food systems. Environ. Pollut. Bioavailab..

[62] Shabanov M.V., Marichev M.S., Minkina T.M., Mandzhieva S.S., Nevidomskaya D.G. (2023). Assessment of the Impact of Industry-Related Air Emission of Arsenic in the Soils of Forest Ecosystems. Forests.

[63] Renco M., Cerevkova A., Hlava J. (2022). Life in a Contaminated Environment: How Soil Nematodes Can Indicate Long-Term Heavy-Metal Pollution. J. Nematol..

[64] Ma Y., Li Y., Fang T., He Y., Wang J., Liu X., Wang Z., Guo G. (2023). Analysis of driving factors of spatial distribution of heavy metals in soil of non-ferrous metal smelting sites: Screening the geodetector calculation results combined with correlation analysis. J. Hazard. Mater..

[65] Gerdelidani A.F., Towfighi H., Shahbazi K., Lamb D.T., Choppala G., Abbasi S., Bari A.S.M.F., Naidu R., Rahman M.M. (2021). Arsenic geochemistry and mineralogy as a function of particle-size in naturally arsenic-enriched soils. J. Hazard. Mater..

[66] Zou Q., Wei H., Chen Z., Ye P., Zhang J., Sun M., Huang L., Li J. (2023). Soil particle size fractions affect arsenic (As) release and speciation: Insights into dissolved organic matter and functional genes. J. Hazard. Mater..

[67] Panthi G., Choi J., Jeong S.-W. (2021). Evaluation of Long-Term Leaching of Arsenic from Arsenic Contaminated and Stabilized Soil Using the Percolation Column Test. Appl. Sci..

[68] Bei Q., Yang T., Ren C., Guan E., Dai Y., Shu D., He W., Tian H., Wei G. (2023). Soil pH determines arsenic-related functional gene and bacterial diversity in natural forests on the Taibai Mountain. Environ. Res..

[69] Chang C., Li F., Wang Q., Hu M., Du Y., Zhang X., Zhang X., Chen C., Yu H.-Y. (2022). Bioavailability of antimony and arsenic in a flowering cabbage-soil system: Controlling factors and interactive effect. Sci. Total Environ..

[70] Frascareli D., Gontijo E.S.J., Silva S.C., Melo D.S., de Castro Bueno C., Simonetti V.C., Barth J.A.C., Carlos V.M., Rosa A.H., Friese K. (2022). Statistical Approaches Link Sources of Sediment Contamination in Subtropical Reservoirs to Land Use: An Example from the Itupararanga Reservoir (Brazil). Water Air Soil Pollut..

[71] Hou T., Filley T.R., Tong Y., Abban B., Singh S., Papanicolaou A.N.T., Wacha K.M., Wilson C.G., Chaubey I. (2021). Tillage-induced surface soil roughness controls the chemistry and physics of eroded particles at early erosion stage. Soil Tillage Res..

[72] Dong J., Zhao W., Shi P., Zhou M., Liu Z., Wang Y. (2023). Soil differentiation and soil comprehensive evaluation of in wild and cultivated Fritillaria pallidiflora Schrenk. Sci. Total Environ..

[73] Antonio D.C., Caldeira C.L., Freitas E.T.F., Delbem I.D., Gasparon M., Olusegun S.J., Ciminelli V.S.T. (2021). Effects of aluminum and soil mineralogy on arsenic bioaccessibility. Environ. Pollut..

[74] Xu N., Zhang F., Xu N., Li L., Liu L. (2023). Chemical and mineralogical variability of sediment in a Quaternary aquifer from Huaihe River Basin, China: Implications for groundwater arsenic source and its mobilization. Sci. Total Environ..

